# Genomic Selection and Genome-wide Association Study for Feed-Efficiency Traits in a Farmed Nile Tilapia (*Oreochromis niloticus*) Population

**DOI:** 10.3389/fgene.2021.737906

**Published:** 2021-09-20

**Authors:** Agustin Barría, John A. H. Benzie, Ross D. Houston, Dirk-Jan De Koning, Hugues de Verdal

**Affiliations:** ^1^ The Roslin Institute and Royal (Dick) School of Veterinary Studies, University of Edinburgh Easter Bush, Midlothian, United Kingdom; ^2^ WorldFish, Bayan Lepas, Malaysia; ^3^ School of Biological Earth and Environmental Sciences, University College Cork, Cork, Ireland; ^4^ Department of Animal Breeding and Genetics, Swedish University of Agricultural Sciences, Uppsala, Sweden; ^5^ CIRAD, UMR ISEM, Montpellier, France; ^6^ ISEM, Univ Montpellier, CNRS, EPHE, IRD, Montpellier, France; ^7^ CIRAD, UMR AGAP Institut, Montpellier, France; ^8^ UMR AGAP Institut, Univ Montpellier, CIRAD, INRAE, Institut Agro, Montpellier, France

**Keywords:** nile tilapia, feed conversion ratio, genomic prediction, SNP array, GWAS, candidate genes, feed intake

## Abstract

Nile tilapia is a key aquaculture species with one of the highest production volumes globally. Genetic improvement of feed efficiency via selective breeding is an important goal, and genomic selection may expedite this process. The aims of this study were to *1*) dissect the genetic architecture of feed-efficiency traits in a Nile tilapia breeding population, *2*) map the genomic regions associated with these traits and identify candidate genes, *3*) evaluate the accuracy of breeding value prediction using genomic data, and *4*) assess the impact of the genetic marker density on genomic prediction accuracies. Using an experimental video recording trial, feed conversion ratio (FCR), body weight gain (BWG), residual feed intake (RFI) and feed intake (FI) traits were recorded in 40 full-sibling families from the GIFT (Genetically Improved Farmed *Tilapia*) Nile tilapia breeding population. Fish were genotyped with a ThermoFisher Axiom 65 K Nile tilapia SNP array. Significant heritabilities, ranging from 0.12 to 0.22, were estimated for all the assessed traits using the genomic relationship matrix. A negative but favourable genetic correlation was found between BWG and the feed-efficiency related traits; −0.60 and −0.63 for FCR and RFI, respectively. While the genome-wide association analyses suggested a polygenic genetic architecture for all the measured traits, there were significant QTL identified for BWG and FI on chromosomes seven and five respectively. Candidate genes previously found to be associated with feed-efficiency traits were located in these QTL regions, including *ntrk3a*, *ghrh* and *eif4e3*. The accuracy of breeding value prediction using the genomic data was up to 34% higher than using pedigree records. A SNP density of approximately 5,000 SNPs was sufficient to achieve similar prediction accuracy as the full genotype data set. Our results highlight the potential of genomic selection to improve feed efficiency traits in Nile tilapia breeding programmes.

## Introduction

One of the key features of aquaculture species compared with terrestrial farmed species is their greater feed efficiency (FE) (Brown, 2006). For example, fish need around six times less feed than cattle to produce the same amount of body mass (de Verdal et al., 2018a). However, feed still remains the primary cost for farmed fish production, and relatively little direct selection for improved feed efficiency has yet been performed for most aquaculture species. Therefore, genetic improvement of FE would enhance the economic sustainability of aquaculture and reduce environmental impacts, including greenhouse gas emissions (Aubin et al., 2009; MacLeod et al., 2020). Since domestication of aquaculture species is relatively young (Houston et al., 2020), selective breeding offers a great potential to improve commercially important traits (FAO, 2019). The benefits of genetic improvement have been illustrated in some key aquaculture species in relation to growth rate and disease resistance, particularly when augmented by genomic tools (see detailed reviews by Gjedrem and Rye, 2018; Fraslin et al., 2020; Houston et al., 2020). However, comparative little direct focus has been placed on feed efficiency in most aquaculture species, most likely due to the challenges of measuring feed intake efficiently and accurately at individual level (de Verdal et al., 2018a).

Nile tilapia (*Oreochromis niloticus*) is the third most produced farmed species globally (FAO, 2020). Farmed across a wide range of production systems, this species is considered a critical protein source for human consumption in undeveloped and developing countries (Miao and Wang, 2020). Recently, a wide variety of genomic tools have been developed for Nile tilapia (reviewed in Yáñez et al., 2020). These genomic tools have facilitated studies to unravel the relationships among improved strains (Hamilton et al., 2020), to detect regions associated with important traits (Cáceres et al., 2019; Yoshida et al., 2019; Taslima et al., 2020; Barria et al., 2021), and to assess the accuracy of prediction of breeding values using genomic selection (GS) models, compared with pedigree-based models (Yoshida et al., 2019; Joshi et al., 2020). However, there is a lack of studies targeting identification of genomic regions associated with FE traits (i.e. those which involve recording of feed intake), or assessing the potential impact of using genomic approaches to improve these traits in a Nile tilapia breeding populations. Previous work has aimed to assess body weight related traits (e.g. harvest weight, head weight, body length, fillet yield), as has been reviewed in Yáñez et al. (2020). Therefore, the aims of this study were to *1*) dissect the genetic architecture of feed-efficiency traits in a Nile tilapia breeding population, *2*) map the genomic regions associated with these traits and identify candidate genes, *3*) evaluate the accuracy of prediction of breeding values using genomic data, and *4*) assess the impact of the SNP density on the genomic prediction accuracies. To the best of our knowledge, this is the first study to assess genomic prediction for feed-efficiency traits in Nile tilapia. Our results highlight the feasibility of including feed-efficiency traits into Nile tilapia breeding programs. Furthermore, they show the potential improvement in breeding value prediction using low density SNP panels compared to using pedigree methods, which can enhance genetic gain and improve production efficiency.

## Materials and Methods

### Nile Tilapia Breeding Population

This study used tissue samples archived by de Verdal et al. (2018b) from their study of genetic parameters of FE related traits in GIFT tilapia. In brief, the fish used in this study were from the Genetically Improved Farmed *Tilapia* (GIFT) breeding program based in Jitra, Malaysia, and managed by WorldFish (Ponzoni et al., 2011) These fish had been selected for increased growth rate for 15 generations in Malaysia at the time the experiment was performed. A total of 40 full-sibling families produced by natural spawning between December 2014 and December 2015 were used for the experimental challenge. All fish were fin clipped (the clip was stored individually in analytical grade ethanol) to provide tissue for DNA analysis at the beginning of the feed intake measurement stage (see next section).

### Feed Conversion Experimental Challenge

Full details of the experimental challenge are given in previous studies, which reported methodology development and quantitative genetic analysis of feed efficiency traits (de Verdal et al., 2017; 2018b). In summary, a total of 1,200 fish (30 fish/family) were used for the feed efficiency experiment. There were four batches assessed during a period lasting from June 2015 to April 2016. For each batch, each full-sibling family was randomly split and transferred into two 200 L aquaria, such that each aquarium had a total of 15 fish. These fish were kept in the aquarium throughout the experimental challenge. To measure the amount of feed consumed daily by each fish, all fish were tagged with a unique two colored T-bar tag (Avery Dennison tags, 25 mm) combination at the dorsal muscle. The experimental challenge consisted of four different stages; 1) adaptation, 2) fasting, 3) feeding and 4) feed intake (FI), lasting 15, 10, 17 and 7 days, respectively. The number of days selected for each stage is described as follows: *1*) Generally 1 week is sufficient for an adaptation period. However, this was increased to 2 weeks as a precaution because some fish could adapt faster than others. The 10 days of fasting 2) represents the longest period permitted while maintaining acceptable fish welfare, while fewer days would probably have a low impact in terms of weight loss. The longest period was ascribed to the feeding stage 3), which allows the fish to recover from the fasting period but also enables successful measurement of compensatory growth, ensuring that this does not impact on the measurements of the feeding and growth during the last stage. Previous results by de Verdal et al. (2017), showed that the repeatability of FI was over 95%, when at least 11 meals were measured. Based on this, we selected 13 meals (7 days) for the current FI period 4).

Body weight was measured at the beginning and end of each stage. Due to aggression between fish, only 1,029 fish were available at the beginning of the FI stage where fish were fed twice a day (07:00 and 13:00), pellet by pellet, and videos of each meal were recorded. Fish received a total of 13 meals during this stage (on the first day fish were weighed in the morning and only received one meal). Uneaten pellets were removed at the end of each meal. The number of pellets consumed by each fish was counted through video analysis. Throughout the experiment, water temperature was fixed at 28°C ± 1°C, while the photoperiod was 12L:12D cycle. The phenotypic sex of each fish was registered by visual observation of the gonads at the end of the FI recording period. The fish were sufficiently developed to show sexual differentiation, but were not yet exhibiting any reproductive behaviour. Mortality was recorded daily throughout the challenge. Thirty two fish died during the FI stage and were not included in the analyses.

### Trait Definitions

As a practical calculation of pellet weight, a total of 500 pellets (16.4 ± 1.76 mg) were weighed, and as the variation in weight per pellet was considered low enough, the average weight of each pellet was used. Thus, the FI in grams for each fish could then be calculated as the sum of pellets consumed during the 13 meals. Body weight gain (BWG) was calculated during the FI stage as the difference between the body weight at the end and at the beginning of this stage. The quotient between these two traits (FI/BWG) was used to estimate the feed conversion ratio (FCR). Finally, residual feed intake (RFI) was estimated as the difference between the amount of feed intake by each fish and the amount predicted (Koch et al., 1963).

Outliers were highlighted using the boxplot. stats function of the R package “stats” (R Development Core Team, 2018) and were not included in the analyses.

### SNP Array Genotyping

Total DNA was extracted from fin clips and genotyped by Identigen (Dublin, Ireland) by using a 65 K Axiom^®^ SNP array (Peñaloza et al., 2020). The raw genotype data were filtered using the Axiom analysis Suite v4.0.3.3. Samples with a dish quality control <0.82 and/or genotype calling rate <0.93 were excluded from further analyses, leaving 801 samples. A total of 53 K SNPs were categorized as PolyHighResolution, and therefore were retained for subsequent analyses. Further quality control using Plink software v1.09 (Purcell et al., 2007) was performed. Specifically, SNPs were excluded for the genomic analyses due to either; *1*) call rate <0.95, *2*) minor allele frequency (MAF) < 0.05 or *3*) deviation from Hardy-Weinberg Equilibrium (p < 1 × 10^−6^). Simultaneously, animals with a call rate <0.95 were also excluded. Finally, to excluded potentially duplicated samples, both of each pair of fish with a proportion of IBD higher than 0.7 were removed from further analyses. Thus, a total of 755 fish and 48,431 SNPs remained after these filters, representing the final data for the SNP array (Table 1.).

**TABLE 1:** Summary statistics for all the phenotyped fish for each feed-efficiency related trait.

Traits^a^	FCR	BWG	RFI	FI
N	726	755	727	735
Min	0.48	0.76	−4.41	1.91
Mean	0.94	9.18	−0.40	8.30
Std Dev	0.20	2.98	1.49	2.28
Median	0.91	8.94	−0.40	8.16
Max	1.55	18.24	4.40	15.22

a FCR: Feed Conversion ratio, BWG: body weight gain (in g); FI: feed intake (in g); RFI: residual feed intake (in g).

To assess the genetic structure within the Nile tilapia population, a principal component analysis (PCA) was performed through PLINK v1.09. The two main components were plotted along the two axes in R.

### Prediction of Breeding Values

Only fish with phenotype and genotype data were used for the pedigree-based BLUP (PBLUP) model, to enable a fair comparison between pedigree and genomic prediction performance. To predict the Estimated Breeding Values (EBVs) and estimate the variance components, the following univariate linear model was used:

y=μ+ Xβ+Za+Wc+e (1)

Where y is the vector of phenotypes, μ is the overall average of phenotypic records, β is the vector of fixed effects accounting for sex and batch, a is the vector of random additive genetic effects, *c* is the vector of random effect associated with the common environmental effects (aquarium effect), *e* is the vector of random residuals errors, whereas X **,** Z and **W** are the incidences matrices for the fixed, genetic and environmental effects, respectively. A normal distribution was assumed for the random genetic, common, and residuals effects, with a mean of zero and the following variance:

$$var\;\left[\begin{array}{c} a\\ c\\ e \end{array}\right]=\;\left[\begin{array}{ccc} A\sigma_{a}^{2} & 0 & 0\\ 0 & I\sigma_{c}^{2} & 0\\ 0 & 0 & I\sigma_{e}^{2} \end{array}\right]$$

Where $\sigma_{a}^{2}$, $\sigma_{c}^{2}$and $\sigma_{e}^{2}$ are the additive genetic, common environmental, and residual variance, respectively, whereas A and **I** are the numerator relationship and identity matrix, respectively. For all the traits, heritability was estimated as follows:

$$h^{2}=\;\frac{\sigma_{a}^{2}}{\sigma_{a}^{2}+\;\sigma_{c}^{2}+\sigma_{e}^{2}}$$

As described by VanRaden (2008), for the genomic BLUP (GBLUP), the numerator A relationship matrix was replaced by the genomic **(G)** relationship matrix. In this case, the vector of random additive genetic polygenic effects *g* has a distribution ∼ N (0, $\sigma_{g}^{2}$).

The single-step GBLUP (ssGBLUP) model was the same as for PBLUP and GBLUP. However, a combined kinship matrix H (Aguilar et al., 2010) which included information from the A and G matrix (Wang et al., 2012), was used. Thus, using data from pedigree and genotype data, the inverse of this H matrix is:

$$H^{-1}=\;A^{-1}+\;\left[\begin{array}{cc} 0 & 0\\ 0 & G^{-1\;}-\;A_{22}^{-1} \end{array}\right]$$

Where $A^{-1}$ and $G^{-1\;}$ are the inverse of the numerator and genomic relationship matrix, respectively, and $A_{22}^{-1}$ is the inverse of the A matrix, but only for the genotyped fish. The estimation of the genetic parameters and models were fitted using the BLUPF90 family program (Misztal et al., 2015).

### Predictive Ability and Cross Validation

The ability to predict the breeding values for the traits using pedigree and genomic approaches was assessed using a fivefold cross-validation approach. To assess the predictive ability among the different models, a total of 100 replicates were used. For each replicate, 80% of fish were used as a training dataset and, for the remaining 20%, the phenotypes were masked and used as the validation dataset. For each model, two cross-validation approaches were applied:

1)Random animal: for each replicate, 80% of all the fish were randomly drawn independently of their family, and used as a training dataset. The remaining 20% of the samples were used as a validation dataset.
2)Random families: for each replicate, 80% of the families were randomly drawn and used as a training set. For the remaining 20% of the families, the phenotype was masked and used as a validation dataset. Thus, breeding value predictions were made in the validation set using phenotypic data from different families.

For each approach, prediction accuracy was calculated as follows:

$$Accuracy=\;\frac{r\left(y_{1},\;\;y_{2}\right)\;}{h}$$

Where $y_{1}$ and $y_{2}$ represents the predicted EBV and the phenotype, respectively, r is the correlation between these both values, and h is the square root of the estimated heritability.

Significant differences in predicted accuracies among models were assessed for each trait using both approaches. A Shapiro-Wilk test was done to evaluate the normality of the distribution of the predicted accuracies. The “rstatix” R package (Kassambara, 2021), was used for a pairwise comparison of the marginal means among models. Finally, the obtained *p* values were adjusted by the number of independent tests.

### SNP Densities

Since the relatively high cost of high density SNP array genotyping may present a barrier to routine genotyping of large number of animals within a population, the efficacy of reduced density SNP panels for predicting breeding values was assessed. A total of 23 subsets of random SNPs were selected from the high density SNP array. These densities included 0.1, 0.3, 0.5, 0.7, 0.9, 1, 2, 3, 4, 5, 6, 7, 8, 9, 10, 20, 30, 40, 50, 60, 70, 80 and 90% of the entire SNP dataset. For each SNP density, 100 replicates of a random animal five-fold cross validation were generated. The GBLUP model fitted for each density is the same as that described for the full genotype data set, and the accuracy was predicted as detailed previously.

### Genome-wide Association Analyses

To identify genomic regions associated with the FE traits, a similar model to that proposed for genomic predictions was applied. However, since random common environmental effect was found to be non-significant, it was not included in the models. The GWAS was performed using the *leaving-one-chromosome-out* (LOCO) approach from the GCTA software v.1.92.2 (Yang et al., 2011). Briefly, a suggestive and genome-wide (α = 1 and 0.05, respectively) Bonferroni significance threshold was used to assess the significance of individual SNPs associated with the assessed traits. The former was first proposed by Lander and Kruglyak (1995), suggesting that at least one false positive marker is expected under the null hypothesis. Thus, a SNP was considered significant at a suggestive or genome-wide level if surpassed the corresponding Bonferroni significance threshold. The SNPs which were not mapped to a specific chromosome of the Nile tilapia reference genome (Conte et al., 2019; Genbank accession number GCA_001858045.3) were assigned to an artificial chromosome “Oni24”.

For the significant QTLs associated with the traits, candidate genes were identified within a 500 Kb window size flanking the associated markers (250 Kb upstream and downstream, respectively) and used as the basis for a literature search for relevant functions connected to feed intake and efficiency. This size was selected based on Cádiz et al., 2020, which used a similar window size for look candidate genes in three commercial Nile tilapia populations.

## Results

### FCR Challenge

Summary statistics for the feed efficiency and growth traits calculated after the trial are shown in Table 1. In brief, the BW of some fish increased by as little as 0.76 g, while others gained up to 18.24 g. In case of the FCR, the mean value was 0.94 ± 0.20, ranging from 0.48 to 1.55. The average RFI and FI were −0.40 ± 1.49 g and 8.30 ± 2.28 g, respectively, ranging from −4.41 to 4.40 g and from 1.91 to 15.22 g, respectively.

### Genetic Parameters

The additive genetic variance estimated through PBLUP models was higher compared with GBLUP and ssGBLUP. The PBLUP model also had the lowest estimates for the environmental and residual variance (Table 2).

**TABLE 2:** Estimated additive genetic ($\sigma_{a}^{2}$ ), common ($\sigma_{c}^{2}$ ), and residual ($\sigma_{e}^{2}$) variance parameters, common environmental effects (c^2^) and heritabilities (h^2^) for feed conversion ratio (FCR), body weight gain (BWG), residual feed intake (RFI) and feed intake (FI). Standard error is shown inside brackets.

Trait	Model^a^	$\sigma_{a}^{2}$	$\sigma_{c}^{2}$	$\sigma_{e}^{2}$	$c^{2}$	$h^{2}$
FCR	PBLUP	7.7E-03	1.7E-03	2.8E-02	0.05 (0.03)	0.21 (0.09)
	GBLUP	4.4E-03	2.4E-03	2.9E-02	0.07 (0.04)	0.12 (0.06)
	ssGBLUP	4.5E-03	2.3E-03	2.9E-02	0.06 (0.04)	0.12 (0.06)
BWG	PBLUP	1.82	0.18	4.60	0.03 (0.03)	0.28 (0.10)
	GBLUP	1.41	0.32	4.78	0.05 (0.03)	0.22 (0.07)
	ssGBLUP	1.46	0.31	4.81	0.05 (0.03)	0.22 (0.07)
RFI	PBLUP	0.96	3.7E-02	1.16	0.02 (0.02)	0.45 (0.12)
	GBLUP	0.37	0.14	1.49	0.07 (0.04)	0.19 (0.07)
	ssGBLUP	0.39	0.13	1.50	0.06 (0.04)	0.19 (0.07)
FI	PBLUP	2.19	2.5E-02	2.02	6.0E-03 (0.02)	0.52 (0.14)
	GBLUP	0.61	0.26	2.91	0.07 (0.04)	0.16 (0.06)
	ssGBLUP	0.64	0.24	2.91	0.06 (0.04)	0.17 (0.07)

a PBLUP: pedigree-based BLUP models; GBLUP: genomic-based BLUP models; ssGBLUP: single-step genomic-based BLUP models.

Heritability estimates using PBLUP, GBLUP and ssGBLUP were significant, and low to moderate in magnitude (Table 2). In all cases, the estimates were higher when a PBLUP model was fitted (ranging from 0.21 to 0.52), while both genomic models had similar estimates for a given trait (ranging from 0.12 to 0.22). No significant differences were found for c^2^ among models.

A high genetic correlation of 0.98 ± 0.03 between FCR and RFI was found, suggesting that both measurements are essentially representations of the same trait (Table 3). Negative but favourable genetic correlations were found between BW, and both FCR (−0.60 ± 0.16) and RFI (−0.63 ± 0.17). However, when pedigree-based data was used, these correlations were not significantly different from zero (Supplementary Table S1).

**TABLE 3:** Estimated genetic correlation with standard error in brackets estimated using GBLUP models among feed conversion ratio (FCR), body weight gain (BWG), residual feed intake (RFI) and feed intake (FI).

	FCR	BWG	RFI	FI
FCR	–	–	–	–
BWG	-0.60 (0.16)	–	–	–
RFI	0.98 (0.03)	−0.63 (0.17)	–	–
FI	0.24 (0.25)	0.61 (0.16)	0.21 (0.23)	–

### Genome-wide Association

**FIGURE 1:**
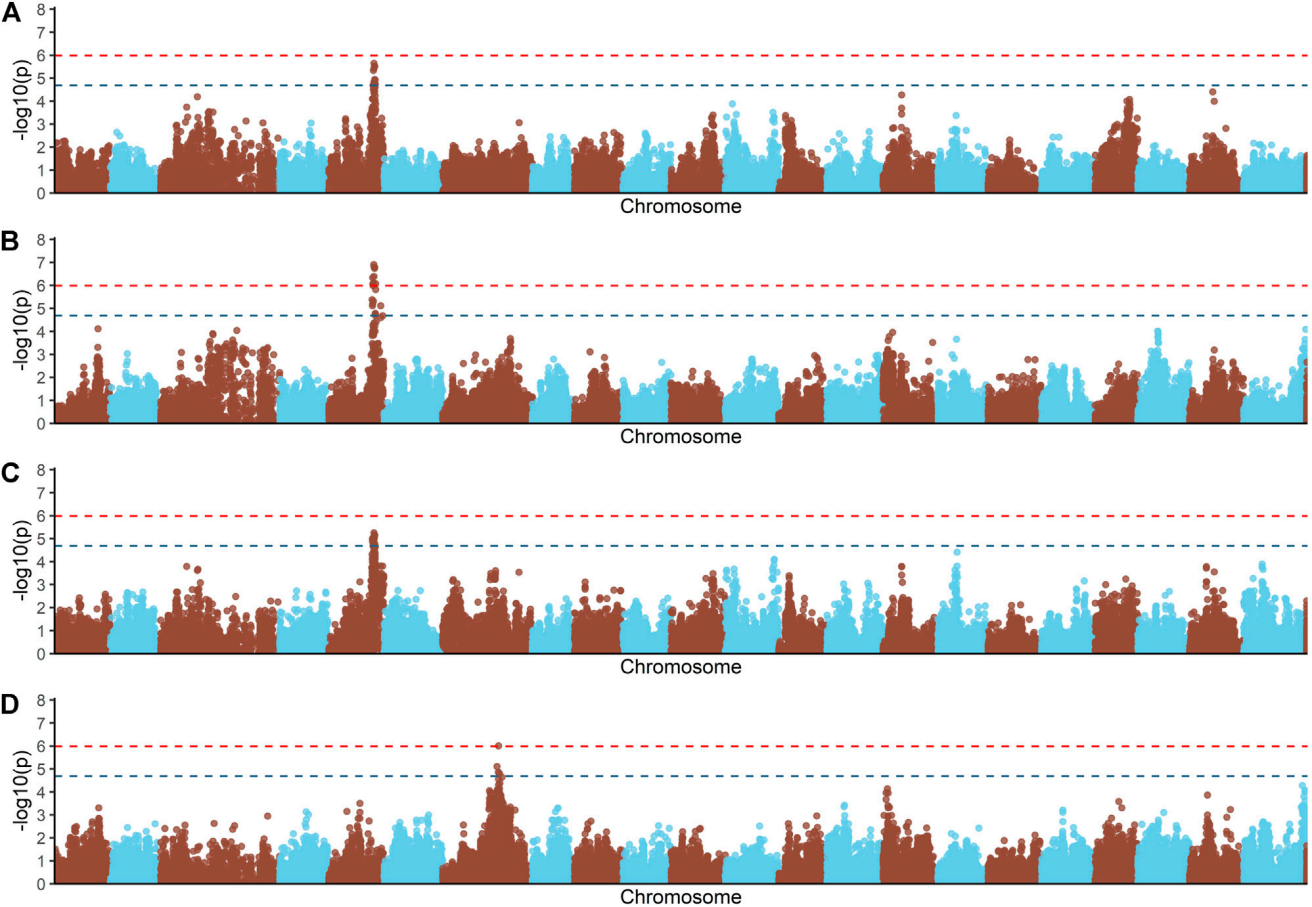
Genome-wide association analysis for feed conversion rate related traits in a Nile tilapia (*Oreochromis niloticus*) breeding population. Genome-wide association analyses for feed conversion ratio **(A)**, body weight gain **(B)**, residual feed intake **(C)** and feed intake **(D)**. Red and blue dashed line represents the genome-wide and suggestive Bonferroni significant threshold, respectively.

Principal components analyses indicated that the main two components accounted together for 15.6% of the total genetic variation (Figure 1S), with no a clear structure evident within the population. There was a suggestive QTL on *Oni5* associated with both FCR and RFI (Figures 1A, C). The same SNPs were associated with both traits, consistent with the high positive genetic correlation between them. A genome-wide significant QTL for BWG (Figure 1B) was identified in the same region of chromosome *Oni5*, supported by a total of 10 SNPs. These ten SNPs were located within a 2.1 Mb window (Table 4). Lastly, there was a single SNP significantly associated with FI on chromosome *Oni7* (Figure 1D). The summary statistics for all the significant SNPs found for BWG and FI together with their detailed locations and allelic information are shown in Table 4.

**TABLE 4:** Information on the genomic location, allelic variants and summary statistics for the genome-wide significant markers associated with feed-efficiency related traits in a Nile tilapia breeding population.

Body weight gain (BWG)					
*Oni* *^a^*	SNP	BP^b^	Minor allele	Major allele	Pval
5	AX-317149233	31.78	A	C	8.7E-07
5	AX-317149515	32.14	G	A	7.8E-07
5	AX-317169164	32.44	C	T	1.2E-07
5	AX-317169167	32.47	G	A	1.6E-07
5	AX-317149783	32.53	C	T	4.1E-07
5	AX-317169548	32.99	T	C	1.8E-07
5	AX-317150097	32.99	A	G	1.7E-07
5	AX-317150157	33.02	G	A	1.7E-07
5	AX-317150877	33.69	A	G	8.5E-07
Feed intake (FI)					
7	AX-317210965	40.23	C	T	9.9E-07

a Number of chromosome on the *Oreochromis niloticus* reference genome.

b Position of the SNP in the chromosome, in million base pairs.

The genes within a 500 Kb window surrounding the most significant SNPs associated with these traits are summarized in Table 5. The *Eukaryotic translation initiation factor 4E* (*eif4e3*) and *growth hormone-releasing hormone* (*ghrh*) were found within the QTL for BWG, *eif4e3* being situated approximately 89 Kbp upstream from the most significant SNP (AX-317169164), whereas *ghrh* is ∼2 Kb upstream of the SNP AX-317150877. In the case of FI, the neurotrophic tyrosine kinase receptor type 3 (*ntrk3*), hyaluronidase 4 (*hyal4*) and *Sus scrofa* sperm adhesion molecule 1 (*spam1*), genes were found within the QTL region.

**TABLE 5:** Genes flanking the significant SNPs associated with feed-efficiency related traits in a Nile tilapia breeding population. Bold genes represent genes with potential impact on feed-efficiency related traits.

SNP	QTL region^b^		Gene names
	Left position	Right position	
Body weight gain (BWG)			
AX-317149064	31.31	31.81	*gnl3l, comt, traip, mon1a, trnar-acg, **bhlhe40, mst1r,** camkv, naaa, sema3f*
AX-317149233	31.53	32.03	*trnar-acg, bhlhe40, **trnt1,** camkv, naaa, sema3f*
AX-317149515	31.89	32.39	*trnt1, ddx4, gpr27, mdfic2, eif4e3, foxp1*
AX-317169164	32.19	32.69	*gpr27, ppp4r2, eif4e3, rybp, gxylt2, foxp1, pdzrn3, shq1*
AX-317169167	32.22	32.72	*gpr27, ppp4r2, eif4e3, rybp, gxylt2, foxp1, pdzrn3, shq1*
AX-317149783	32.28	32.78	*gpr27, ppp4r2, **eif4e3,** rybp, gxylt2, foxp1, pdzrn3, shq1*
AX-317169548	32.74	33.24	*chl1*
AX-317150097	32.74	33.24	*chl1*
AX-317150157	32.77	33.27	*chl1*
AX-317150877	33.44	33.94	*cdk5rap1, **ghrh,** ift52, srsf6, **rpn2,** mybl2, **acot8***
Feed Intake (FI)			
AX-317210965	39.98	40.48	*akap13, ska1, agbl1, ntrk3a, pard6a, wasla, hyal6, hyal4, spam1, gpr37a, pot1, grm8b, acot4*

### Accuracy of Breeding Value Predictions

**FIGURE 2:**
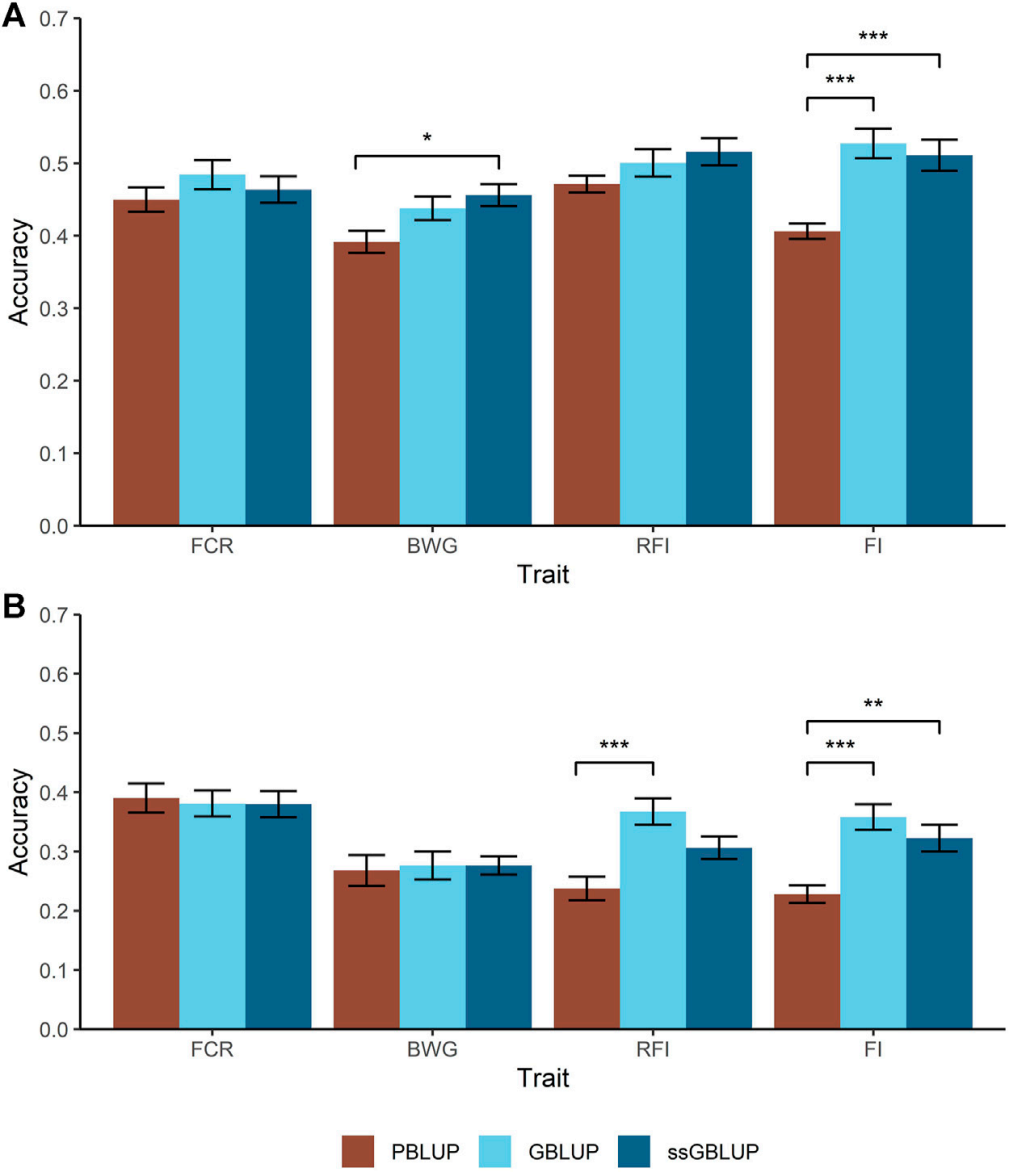
Predicted accuracies comparison for the feed-efficiency related traits in a Nile tilapia (*Oreochromis niloticus*) breeding population. Accuracies predicted through pedigree-based BLUP (PBLUP), genomic BLUP (GBLUP) and single-step GBLUP (ssGBLUP) for feed conversion ratio (FCR), body weight gain (BWG), residual feed intake (RFI) and feed intake (FI) using a random animal **(A)** and random family **(B)** cross-validation approach. The standard error for each trait and method is represented by the black bars. * = *p* < 0.05; ** = *p* < 1 × 10^−3^; *** = *p* < 1 × 10^−5^.

Two different cross-validation approaches were tested to estimate prediction accuracies; a random individual and a random family approach. For the former, higher accuracies were achieved for BWG and FI when genomic data were used, with an increase of 34% compared with PBLUP (Figure 2A). However, in case of BWG this increase is only observed when the A and G matrix were combined (ssGBLUP).

As expected, all the models resulted in lower prediction accuracies when a random family approach was used, compared with the random animal approach. Furthermore, two main trends were observed. Firstly, for FCR and BWG, there were no significant differences in prediction accuracies using any of the models incorporating genomic data (Figure 2B). Secondly, the highest accuracies were achieved using GBLUP, representing an increase in 33 and 26% for RFI and FI respectively over PBLUP, while ssGBLUP gave intermediate prediction accuracies for FI.

### Impact of SNP Density on Predicted Accuracies

**FIGURE 3:**
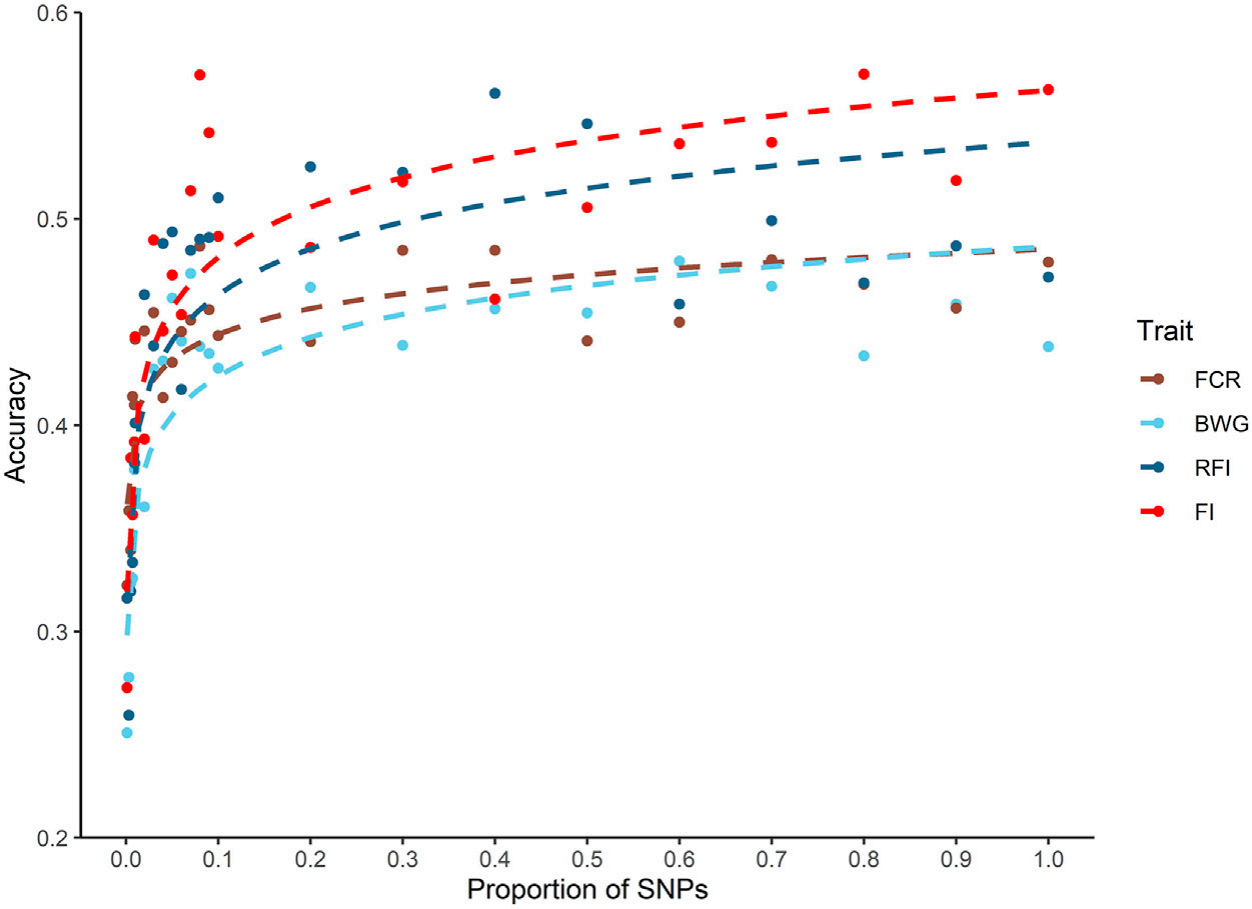
Genomic prediction accuracies using different SNP densities for feed-efficiency related traits in a Nile tilapia (*Oreochromis niloticus*) breeding population. Predicted accuracies achieved using different SNP low density panels for feed conversion ratio (FCR), body weight gain (BWG), residual feed intake (RFI) and feed intake (FI) using a genomic BLUP (GBLUP) method.

A decrease in the estimated heritabilities were observed with the low density panels (data not shown). Therefore, for the calculation of prediction accuracy at all SNP densities, a single heritability estimated with all the available markers was used, as is considered the most accurate estimate (Kriaridou et al., 2020). Only a slight decrease in the prediction accuracies was observed across all traits when up to 10% of the SNP density was used (5 K SNPs). When SNP density fell below 5K SNPs, the breeding value prediction accuracy was lower for all the traits (Figure 3).

## Discussion

The current study generated genome-wide SNP genotype data from tissue samples collected from a previous study in which genetic parameters for FE traits were estimated in a Nile tilapia breeding population (de Verdal et al., 2018b). Through quantitative genetic analyses and using pedigree data with information from up to 15 generations, significant genetic variation was detected for all the assessed traits, FCR, BWG, RFI and FI. The current study integrated the phenotypic dataset with genome-wide SNP information from recently developed SNP array for Nile *Tilapia* (Peñaloza et al., 2020), to estimate genetic correlations and genomic heritabilities using almost 50 K SNP markers segregating throughout the Nile tilapia genome. The same data were used to dissect the genetic architecture of feed-efficiency traits using a GWAS. Finally, the dataset was used to assess and compare genomic prediction accuracies using different statistical models, and assess the optimal SNP marker density for achieving maximal prediction accuracy.

### Heritability and Genetic Correlations

Genetic parameters estimates for FE traits are relatively rare in aquaculture species, but have been extensively assessed in livestock species with significant heritabilites detected, ranging from 0.13 up to 0.84 (de Verdal et al., 2011; Case et al., 2012; Freetly et al., 2020; Tortereau et al., 2020; Marchesi et al., 2021). The studies on aquaculture species have also detected moderate to high heritabilities (0.57–0.71) for average daily gain (ADG), daily feed intake (DFI), feed efficiency ratio (FER) and RFI in a breeding Pacific white shrimp (*Litopenaeus vannamei*) population (Dai et al., 2017). Furthermore, Kause et al. (2006) found significant heritabilities for growth in rainbow trout (*Oncorhynchus mykiss*), but not for FI, whereas Dvergedal et al. (2019) found significant heritabilities ranging from 0.18 to 0.45 for several feed efficiency traits in Atlantic salmon, such as weight gain, residual weight gain, and stable isotope profile in food. Furthermore, de Verdal et al. (2018b) estimated significant heritability (0.32–0.65) for FE traits in the current Nile tilapia population. Slightly lower heritabilities for the same traits were found in the current study, which could be explained by the ∼25% fewer fish being included in the analyses.

A moderate genetic correlation was found between BWG and both FCR and RFI, when the genomic relationship matrix was used. This favourable and significant correlation suggests that selection for BWG (a simple trait to measure) would result in favourable correlated responses for feed efficiency traits within the current population. Although these results are in agreement with previous studies in sea bass (*Dicentrarchus labrax*) and rainbow trout (Kause et al., 2006, 2016; Silverstein, 2006; Besson et al., 2019), they contrast with those estimated by de Verdal et al. (2018b) who found no significant correlations. This cannot be attributed to differences in sample size, since correlations estimated using only pedigree data in the present study (Supplementary Table S1) were in accordance with de Verdal et al. (2018b). It is challenging to explain this discrepancy between the pedigree and the genomic data. It can be hypothesized that using genomic data, the true relationship between fish within each family were estimated in the genomic relationship matrix, exploiting two different levels of genetic variation: at inter and intra-family level. The latter is not included in the pedigree-based analyses. Therefore, using PBLUP, part of the additive effect went to the common environmental effect since families were not mixed in each aquarium, and so it was only possible to assess the differences between the families. In case of the genomic analyses, the genetic relationships between fish are more accurately estimated by estimating the random recombination during meiosis.

### Genome-wide Association and Candidate Genes

With few exceptions (Houston et al., 2008; Moen et al., 2009; Boison et al., 2019; Sinclair-Waters et al., 2020; Barria et al., 2021), commercially important traits in aquaculture are underpinned by a polygenic genetic architecture (Tsai et al., 2015; Barría et al., 2018; Gutierrez et al., 2018; Palaiokostas et al., 2018; Mohamed et al., 2019; Aslam et al., 2020; Lu et al., 2020). This genetic architecture is also typical of FE traits in several breeding populations of livestock (Yuan et al., 2015; Higgins et al., 2018; Fu et al., 2020; Marchesi et al., 2021). Gutierrez et al. (2015) and Dvergedal et al. (2020) also found a polygenic architecture for these traits in Atlantic salmon. Whereas in the Crucian carp (*Carassius auratus*), several QTLs were detected for ADG, feed conversion efficiency and feed intake (Pang et al., 2017). Yoshida and Yáñez (2021) observed a polygenic architecture for ADG using a multi-trait GWAS approach in a Nile tilapia breeding population. Our results are in agreement with these previous studies, suggesting a polygenic architecture for feed efficiency traits in this current GIFT Nile tilapia breeding population. No major QTL were detected in the current study, but several QTLs were detected which explained a minor proportion of genetic variation for the traits. Also, the possibility that some regions impacting the traits have not been covered by our genetic markers cannot be discarded.

The understanding of the interrelationship process among FE traits at the molecular level could provide new insights into the biological pathways involved and their genetic regulation. To date, a wide range of pathways have been associated with these traits, including forebrain development and neuron differentiation, hormone and growth factors, gluconeogenesis, lipogenesis, epithelial cell differentiation and hematopoietic cell lineage (McKenna et al., 2018; Li et al., 2019; Fu et al., 2020; Lindholm-Perry et al., 2020; Marchesi et al., 2021).

Among the genes flanking the significant markers located on *Oni5* and associated with BWG, *ghrh* was highlighted as one of the most important candidate genes given its strong association with growth rate through stimulation of growth hormone (GH). Fish and higher vertebrates growth is partly controlled by the GH/insulin-like growth factor-I (IGF-I) axis, which is also involved in the regulation of several physiological process in fish such as lipid and protein metabolism, immune function and feeding behaviour (Albalat et al., 2005; Kawauchi and Sower, 2006). For example, the modulation of genes related with the GH/IGF-I axis helps the fish to cope with fasting periods, by diverting the energy otherwise used for growth to other essential physiological processes (Wood et al., 2005; Gabillard et al., 2006). Although the relationship between growth and IGF-I is affected by several endogenous and exogenous factors, varying across seasons and productive cycles (Beckman, 2011), reliable associations have been shown in Mediterranean fishes as sea bass and gilthead sea bream (Company et al., 2001). In the case of tilapia, this association has also been found (Vera Cruz et al., 2006), where a GH-overexpressed transgenic line of Nile tilapia showed significantly higher weight and length than the wildtype (Eppler et al., 2007). Furthermore, in *O. mossambicus,* a recovery in plasma IGF-I levels during feeding stage after fasting, was associated with an increase in weight gain (Fox et al., 2009), and an administration of IGF-I and IGF-II has been shown to stimulate the growth rate (Chen et al., 2000).

Another interesting candidate gene, *eif4e3,* was located close to the most significant SNP for BWG. This gene belongs to the EIF4E family of proteins, which is associated with cell growth through protein synthesis and immune response (Piron et al., 2010). Previous work by Sun et al. (2016) showed significant differential expression of this gene, in *Hulong groupe* (*Epinephelus fuscogutatus x Epinephelus anceolatus*), a hybrid fish with an increased growth rate compared with their parental species.

It’s well known the role of the brain-gut axis on the food intake regulation and nutrient metabolism (Reyer et al., 2015). For example, in zebrafish, modulation of brain activity through feeding and food availability has been reported, as well as an increase in feed intake after injection of a neurotrophic factor (Blanco et al., 2020). The gene *Neurotrophic Tyrosine Kinase Receptor Type 3*, *ntrk3*, the ligand of *neurotrophin* 3 (*ntf3*) may act as a modulator of feeding and satiety in mice and is associated with eating disorders in humans (Mercader et al., 2008). In the current study, this gene is located ∼200 Kb from the significant SNPs for FI in *Oni07*, potentially playing a role on the amount of feed intake within the Nile tilapia population. In agreement with a study in another farmed species, *spam1* and *hyal4*, both genes found within the significant QTL for FI, have been previously reported as associated with FI in Landrace pigs. The authors suggest a role of these genes in fat synthesis and in lipid associated pathways, such as transport and metabolism (Fu et al., 2020). This could be explained by the fact that the amount of lipids plays a key role in the control of feed intake, as has been reported in livestock species (Harvatine and Allen, 2006; Kuhla et al., 2016), mainly by acting on the hypothalamic signals regulating feed consumption and energy expenditure (Allen et al., 2005; Relling et al., 2010).

### Genomic Prediction and Impact of SNP Density

Since several factors such as heritability, linkage disequilibrium, population size and genetic architecture underlying traits can impact the accuracy of the genomic predictions (Morgante et al., 2018), it is necessary to compare the performance of different models when a trait is analyzed for the first time within a specific population. To our knowledge this is the first study to assess genomic predictions for feed-efficiency traits in Nile tilapia. Based on the GWAS results, the models used to predict the EBV accuracies were assumed to have a genetic variance controlled by thousands of markers with small effect (infinitesimal model).

The fact that there was no significant differences between the results for the GBLUP and ssGBLUP models for any of the traits and cross-validation approaches is likely due to the fact that only genotyped fish were included into the models. Therefore, the increase in the estimated relationship among fish due to the A and $A_{22}$ matrices (and hence difference in the predicted accuracy of the EBV) is minimal, and most of the relationship is captured by the G matrix.

The observed differences in the EBV accuracies between the two cross-validation approaches (random versus family approaches) are likely to be explained by the genetic distance between fish from the training and validation groups. It has been shown that predicting EBV using data from fish from different full-sib families (i.e. random-family approach) affects mainly the pedigree-based model and traits that are not possible to measure on the candidate themselves (Tsai et al., 2016; Palaiokostas et al., 2019; Joshi et al., 2020). However, by including genomic data it is possible to exploit both inter- and intra-family genetic variation, achieving more accurate relationship assessments between individuals and therefore higher EBV accuracies (Garcia et al., 2018; Palaiokostas et al., 2018; Vallejo et al., 2019; Yoshida et al., 2019). Therefore, within a breeding programme, it would be preferable to ensure that measurements of FE traits were taken on training populations including close relatives, including full siblings, or the selection candidates. It should also be noted that FE measurements could potentially be taken on candidates themselves, but may be challenging for reasons of practicality and cost.

The impact of SNP density on predicted accuracies has been studied thoroughly for a wide variety of traits and species in aquaculture (Yoshida et al., 2019; Kriaridou et al., 2020; Tsairidou et al., 2020; Al-Tobasei et al., 2021; Vu et al., 2021). The results of these studies highlight the potential to significantly reduce SNP density without affecting the accuracy of prediction. Interestingly, Kriaridou et al. (2020) found an optimal SNP density of approximately 2 K SNPs for genomic prediction accuracy in different traits and species, suggesting a low impact of the genome size, genetic architecture, and family structure. The current study highlights that on average approximately 5 K is the minimal SNP density required to achieve similar accuracies to that of the 50 K SNP array for the tilapia population investigated. Thus, breeding programs could potentially use low density SNP markers (typically cheaper than high density panels) to achieve higher accuracies than using pedigree-based models, potentially increasing genetic gain in a cost-efficient manner.

## Conclusion

Significant heritabilities were confirmed for feed-efficiency traits in a Nile tilapia breeding population, highlighting that genetic improvement is feasible. A negative, but favourable, genetic correlation was detected between BWG and FCR using the genomic data, suggesting selection for BWG may indirectly improve FCR. The traits were polygenic, suggesting the genomic selection may be the most effective route to incorporating genotype data into selection decisions. Genomic prediction markedly outperformed pedigree-based prediction, and this was the case even for relatively low density SNP markers. This study therefore highlights the potential for genomic selection to improve feed efficiency traits in Nile tilapia breeding programmes.

## Data Availability

The datasets generated for this study can be found here: doi:10.6084/m9.figshare.16573118.
