# Supplementary figures and images for: Genomic Selection and Genome-wide Association Study for Feed-Efficiency Traits in a Farmed Nile Tilapia (Oreochromis niloticus) Population
Source: Front Genet. 2021 Sep 20;12:737906. doi: 10.3389/fgene.2021.737906 (PMC8488396; doi:10.3389/fgene.2021.737906)

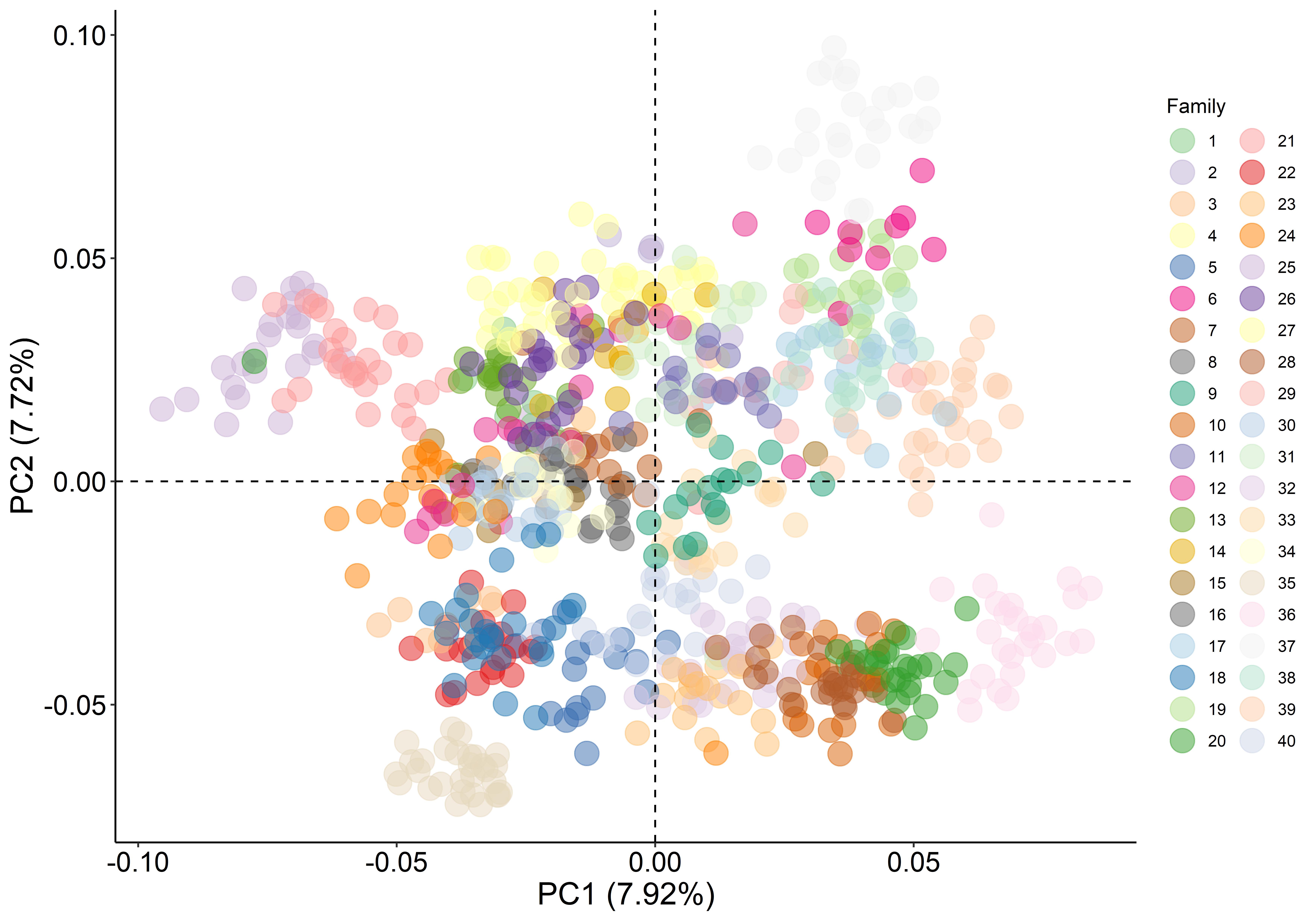

Supplement: Supplementary file 1 [file Image1.JPEG]
